# Glycerol kinase enzyme is a prognostic predictor in esophageal carcinoma and is associated with immune cell infiltration

**DOI:** 10.1038/s41598-024-54425-x

**Published:** 2024-02-16

**Authors:** Fei Ying, Xuyong Chen, Lihong Lv

**Affiliations:** Department of Gastroenterology, Xianju People’s Hospital, NO.53 North East Road, Xianju County, Taizhou, Zhejiang Province China

**Keywords:** GK, Esophageal carcinoma, Prognosis, Immune infiltration, Oesophageal cancer, Risk factors, Gene expression

## Abstract

The influence of lipid metabolism on tumorigenesis and progression has garnered significant attention. However, the role of Glycerol Kinase (GK), a key enzyme in glycerol metabolism, in Esophageal Carcinoma (ESCA) remains unclear. To further elucidate the relationship between GK and ESCA, we investigated GK expression levels using database information. Controlled studies employing immunohistochemistry were conducted on clinical ESCA tumor samples and normal specimens, confirming GK’s elevated expression in ESCA. Analysis of The Cancer Genome Atlas (TCGA) data via Kaplan–Meier (KM) survival plots revealed that increased GK expression correlates with poorer ESCA patient outcomes, particularly in overall survival (OS) and disease-specific survival (DSS). Multiple regression analysis indicated that elevated GK expression is an independent risk factor affecting ESCA prognosis. Statistical analysis of prognostic data from clinical samples further corroborated this finding. Moreover, there appears to be a significant correlation between GK expression and immune infiltration, specifically involving certain T and B lymphocytes. In conclusion, elevated GK expression in ESCA is strongly linked to poor prognosis and increased immune cell infiltration, highlighting its potential as an independent prognostic biomarker and a viable therapeutic target.

## Introduction

Esophageal Carcinoma (ESCA),according to 2020 data ranks as one of the most prevalent malignancies worldwide, occupying the seventh position in incidence (604,000 new cases annually), and the sixth in mortality (544,000 deaths per year)^1^. Notably, the highest regional incidence rates are observed in East Asia, particularly among the Chinese population^1^. Unlike Europe and the United States, where esophageal adenocarcinoma (EA) is the predominant pathological type, esophageal squamous cell carcinoma (ESCC) is more prevalent in China^1,2^. There has been a significant improvement in patient outcomes due to the multidisciplinary and comprehensive treatment models in recent years. The five-year survival rate for patients treated in early stages can exceed 90%, in stark contrast to less than 30% for those with advanced-stage disease^3^. This disparity underscores the importance of early diagnosis and treatment in improving patient prognosis. However, the identification of reliable tumor markers for diagnosis and prognosis in ESCA remains limited.

Glycerol kinase (GK), a protein-coding gene, is expressed in various organs, including the esophagus and liver, with its highest expression in the kidney. The protein encoded by the GK gene plays a crucial role in glycerol uptake and metabolism, catalyzing the phosphorylation of glycerol using adenosine triphosphate (ATP) to produce adenosine diphosphate (ADP) and glycerol-3-phosphate (G3P). This process is essential for the synthesis of glycerolipids, proteins, and glucose. Mutations in this gene are associated with Glycerol Kinase Deficiency (GKD), a condition characterized primarily by hypertriglyceridemia and hypoglycemia^4,5^. Additionally, patients with complex GKD exhibit a wide range of symptoms, including congenital adrenal dysplasia, hyperglyceridemia, and progressive pseudohypertrophic myotonic dystrophy, which can be controlled by glucocorticoid supplementation^4,5^.

Extensive research has established GK’s importance in maintaining normal physiological processes. Rahib et al.’s analysis confirmed GK's direct and indirect connections with lipid and carbohydrate metabolism, insulin signaling, and insulin resistance genes, underscoring its potential role in insulin resistance and type 2 diabetes^6^. Furthermore, GK knockout in mice resulted in neonatal mortality^7^. The role of lipid metabolism-related enzymes in cancer development has received much attention recently, with numerous studies indicating elevated levels of these enzymes in tumor tissues, implicating them in tumor development and prognosis^8,9^. However, the relationship between the GK gene and esophageal ESCA remains unexplored. Given its critical role in regulating glycerol uptake and metabolism, investigating the GK gene's link to the onset and prognosis of ESCA is both important and meaningful.

This study employed various published databases and clinical samples to investigate the difference in GK expression between tumor and normal tissues in ESCA patients. We also examined the relevance of GK expression in terms of prognosis, mutation, methylation, and gene enrichment analysis to uncover potential pathogenic mechanisms. Given the rising importance of immunotherapy in treating advanced diseases and its potential to improve ESCA prognosis^10^, particularly focusing on tumor-infiltrating lymphocytes (TILs), known for their critical role in immune responses^11^. We explored the correlation between GK expression and immune cell infiltration in ESCA. Our findings indicate that GK is a significant biomarker for prognosis and immune infiltration in ESCA, playing a vital role in its pathogenesis.

## Results

### GK expression is up-regulated in ESCA

To investigate the expression level of the glycerol kinase (GK) gene in tumor tissues, we first analyzed GK levels across various tumor types compared to normal tissues using the TIMER database. The results revealed that GK expression was notably upregulated in several cancers, including breast cancer, esophageal squamous cell carcinoma (ESCA), and gastric cancer, while it was significantly downregulated in others, such as colon cancer, lung squamous cell carcinoma, and thyroid carcinoma (Supplementary Fig. 1A).

**Figure 1:**
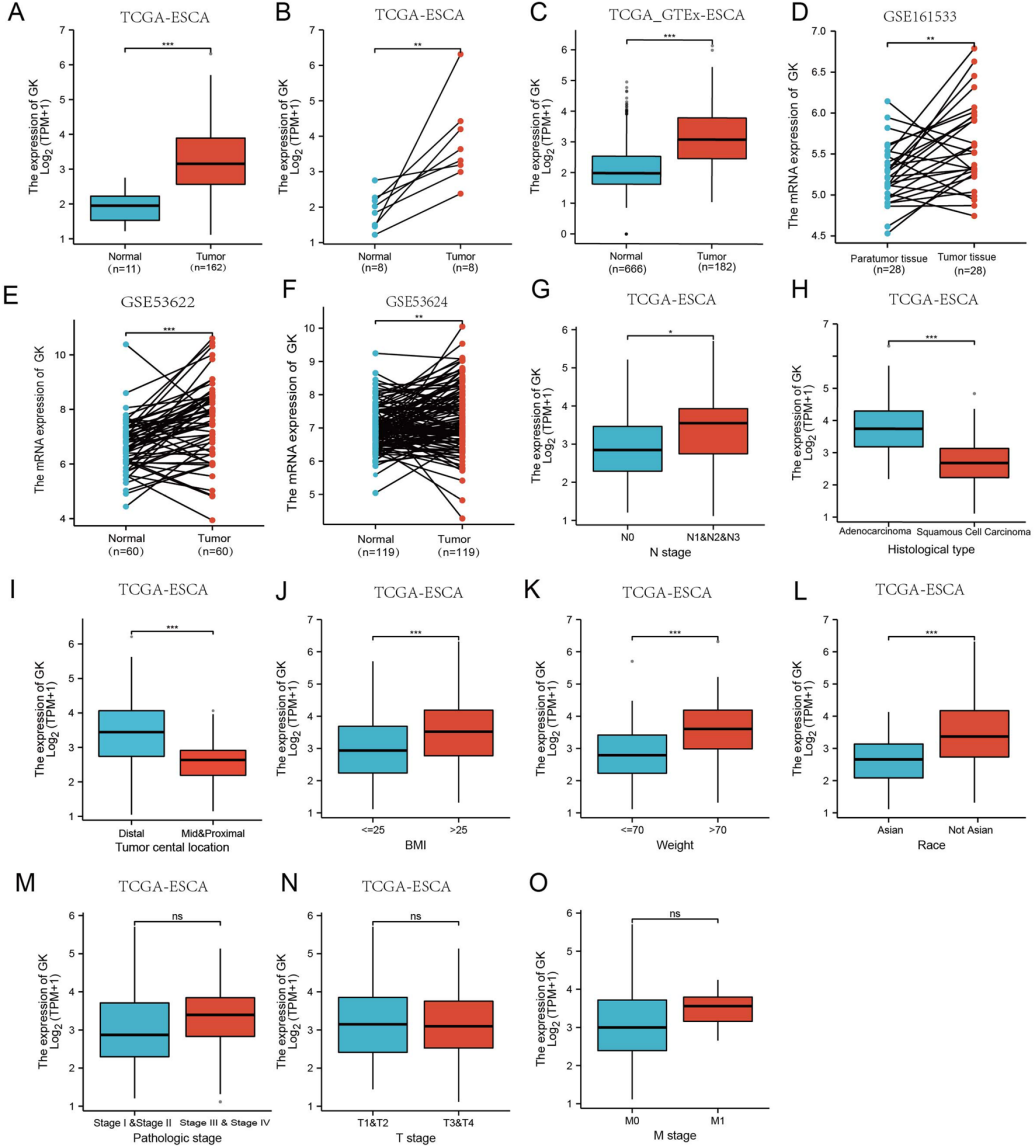
Expression patterns of glycerol kinase (GK) mRNA in esophageal cancer (ESCA) from TCGA, TCGA_GTEx, and GEO Databases. (**A**) GK expression in ESCA versus normal tissues analyzed using the TCGA database. (**B**) Paired GK expression in ESCA and adjacent normal tissues via the TCGA database. (**C**) Comparative analysis of GK expression in ESCA and normal tissues based on the TCGA_GTEx database. (**D**) Paired expression analysis of GK in ESCA versus normal tissues across GSE20916, GSE53622,and GSE53624 datasets. (**G**–**O**) Correlation of GK expression with clinicopathological features in ESCA using TCGA data: N stage (**G**), histological type (**H**), tumor center location (**I**), body mass index (BMI) (**J**), weight (**K**), race (**L**), pathologic stage (**M**), T stage (**N**), M stage (**O**). Statistical significance indicated as: *p < 0.05; **p < 0.01; ***p < 0.001; ‘ns’ denotes no significant difference.

To delve deeper into GK gene expression in ESCA, we utilized the TCGA database to analyze clinical data from 162 ESCA samples and 11 adjacent non-tumor samples. This analysis indicated a significant upregulation of the GK gene in ESCA (p < 0.001, Fig. 1A). Further investigation was conducted on 11 ESCA samples and their matched adjacent non-tumor samples, which corroborated the higher expression of GK in ESCA tissues (p < 0.01, Fig. 1B), aligning with the TIMER database findings.

To expand our control group, we included 666 cases from TCGA_GTEx, further validating the heightened GK expression in ESCA tissues (p < 0.001, Fig. 1C). This finding was supported by data from GSE161533 (p < 0.01, Fig. 1D), GSE53622 (p < 0.01, Fig. 1E), and GSE53624 (p < 0.05, Fig. 1F), sourced from the GEO database. Collectively, these analyses highlight a significant upregulation of GK in ESCA, indicating its potential role in this cancer's pathology.

### Association of high GK expression with clinicopathological characteristics in ESCA patients

We classified ESCA samples from the TCGA database into two groups according to their GK expression levels and summarized their clinical characteristics in Table 1. The analysis identified significant differences between these groups in T stage, DSS events, OS events, pathological type, tumor location, and race (p < 0.05).

**Table 1:** Association of glycerol kinase (GK) expression with clinicopathological parameters in patients with esophageal carcinoma (ESCA).

Characteristic	Low expression of GK	High expression of GK	p
n	81	81	
T stage, n (%)			**0.037**
T1	8 (5.5%)	19 (13.1%)	
T2	24 (16.6%)	13 (9%)	
T3	41 (28.3%)	36 (24.8%)	
T4	2 (1.4%)	2 (1.4%)	
N stage, n (%)			0.211
N0	40 (27.8%)	26 (18.1%)	
N1	27 (18.8%)	36 (25%)	
N2	4 (2.8%)	5 (3.5%)	
N3	3 (2.1%)	3 (2.1%)	
M stage, n (%)			0.144
M0	67 (51.9%)	54 (41.9%)	
M1	2 (1.6%)	6 (4.7%)	
Pathologic stage, n (%)			0.173
Stage I	7 (4.9%)	9 (6.3%)	
Stage II	42 (29.6%)	27 (19%)	
Stage III	24 (16.9%)	25 (17.6%)	
Stage IV	2 (1.4%)	6 (4.2%)	
Histological type, n (%)			** < 0.001**
Adenocarcinoma	19 (11.7%)	61 (37.7%)	
Squamous Cell Carcinoma	62 (38.3%)	20 (12.3%)	
Tumor cental location, n (%)			** < 0.001**
Distal	42 (26.1%)	71 (44.1%)	
Mid	32 (19.9%)	10 (6.2%)	
Proximal	6 (3.7%)	0 (0%)	
Gender, n (%)			1.000
Female	11 (6.8%)	12 (7.4%)	
Male	70 (43.2%)	69 (42.6%)	
DSS event, n (%)			**0.020**
Alive	65 (40.4%)	50 (31.1%)	
Dead	16 (9.9%)	30 (18.6%)	
OS event, n (%)			**0.010**
Alive	57 (35.2%)	40 (24.7%)	
Dead	24 (14.8%)	41 (25.3%)	
Race, n (%)			** < 0.001**
Asian	29 (20.1%)	9 (6.2%)	
Black or African American	5 (3.5%)	1 (0.7%)	
White	42 (29.2%)	58 (40.3%)	
BMI, n (%)			**0.008**
< = 25	51 (33.3%)	33 (21.6%)	
> 25	26 (17%)	43 (28.1%)	
Age, n (%)			1.000
< = 60	42 (25.9%)	41 (25.3%)	
> 60	39 (24.1%)	40 (24.7%)	
Age, meidan (IQR)	60 (53, 70)	60 (54, 73)	0.668

Notes: In the table, values presented in bold indicate statistical significance (p value < 0.05).

T, topography distribution; N, lymph node metastasis; M, distant metastasis; DSS, disease special survival; OS, overall survival; BMI, body mass index.

Furthermore, to probe into the association between high GK expression and clinical characteristics, we reorganized the ESCA data from the TCGA database by different clinical and pathological types. This analysis indicated that higher N-stage GK expression was more prevalent (N0 vs. N1/N2/N3) (p < 0.05, Fig. 1G). GK expression was higher in esophageal adenocarcinoma (EA) compared to esophageal squamous cell carcinoma (ESCC) (p < 0.001, Fig. 1H). Tumor cental location in distal has higher GK expression than location in mid and proximal (p < 0.001, Fig. 1I). Additionally, patients with high BMI, higher weight, and non-Asian ethnicity exhibited elevated GK expression levels (Fig. 1J–L). In contrast, no significant differences were observed in advanced pathological stages (stage I/II vs. III/ IV), T-stage (T1/T2 vs. T3/T4), and M-stage (M0 vs. M1) (Fig. 1M–O)**.**

### The relationship between high GK expression and survival prognosis of ESCA

Univariate Cox regression analysis indicated that N stage, M stage, pathological stage, GK gene expression level, and treatment efficacy significantly influenced the prognosis of ESCA patients (all p values < 0.05, Table 2). Multifactorial regression analysis further revealed that N-stage (HR = 23.368, 95% CI = 3.398–160.699, p = 0.001), M-stage (HR = 161.261, 95% CI = 7.515–3460.457, p = 0.001), and GK gene expression level (HR = 2.320, 95% CI = 1.049–5.132, p = 0.038) were independent risk factors affecting ESCA patient prognosis (Table 2).

**Table 2:** Univariate and Multivariate analysis of clinicopathological factors correlating with overall survival (OS) in patients with esophageal carcinoma (ESCA).

Characteristics	Total (N)	Univariate analysis		Multivariate analysis	
		Hazard ratio (95% CI)	p value	Hazard ratio (95% CI)	p value
T stage (T3 & T4 VS. T1 & T2)	145	1.312 (0.756–2.277)	0.334		
N stage (N1 & N2 & N3 VS. N0)	144	2.970 (1.606–5.493)	** < 0.001**	23.368 (3.398–160.699)	**0.001**
M stage (M1 VS. M0)	129	5.075 (2.312–11.136)	** < 0.001**	161.261 (7.515–3460.457)	**0.001**
Gender (Male VS. Female)	162	2.306 (0.922–5.770)	0.074	1.004 (0.112–8.999)	0.997
Pathologic stage (Stage III & IV VS. Stage I & II)	142	3.223 (1.807–5.747)	** < 0.001**	0.286 (0.062–1.313)	0.108
Age (> 60 VS. < = 60)	162	0.831 (0.506–1.365)	0.466		
Histological type (SCC VS. Adenocarcinoma)	162	0.875 (0.526–1.455)	0.607		
Primary therapy outcome (CR VS. PD & SD & PR)	94	0.336 (0.144–0.784)	**0.012**	7.152 (0.963–53.142)	0.055
GK (High VS. Low)	162	1.289 (1.004–1.655)	**0.046**	2.320 (1.049–5.132)	**0.038**

Notes: Values presented in bold indicate statistical significance (p value < 0.05).

T, topography distribution; N, lymph node metastasis; M, distant metastasis; SCC, squamous cell carcinoma; CR, complete response; PD, progressive disease; SD, stable disease; PR, partial response.

**Figure 2:**
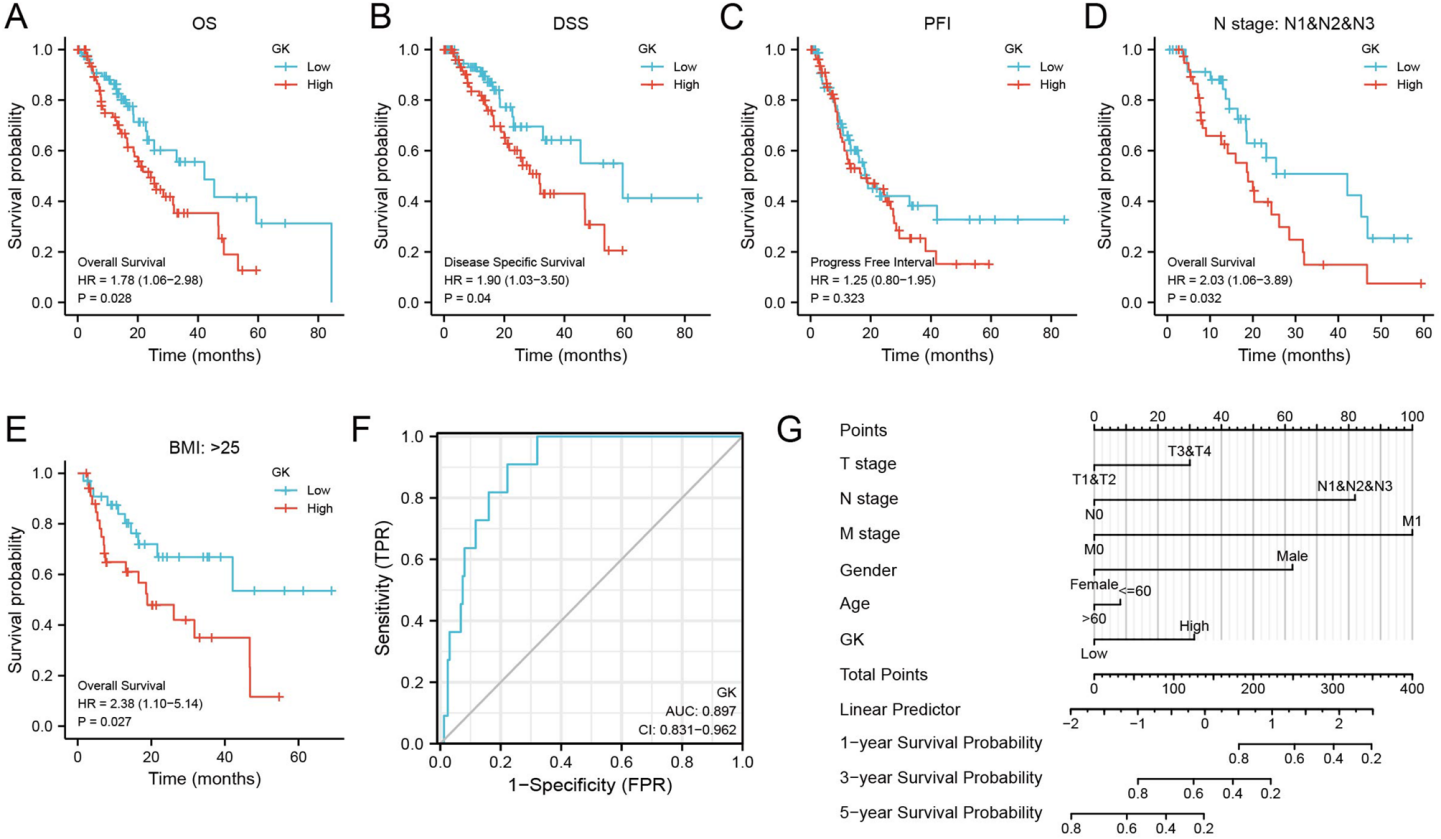
Prognostic and diagnostic value of glycerol kinase (GK) in the TCGA-ESCA Cohort. (**A**–**C**) Kaplan–Meier (KM) survival curve analysis illustrating overall survival (**A**), disease-free survival (**B**), and disease-specific survival (**C**) in ESCA patients. (**D**, **E**) Subgroup KM survival analysis for N1, N2, and N3 stages (**D**) and Body Mass Index (BMI) (**E**), based on the TCGA database. (**F**) Receiver Operating Characteristic (ROC) curve analysis of GK expression in ESCA and adjacent normal tissue. (**G**) A nomogram for predicting 1-year, 3-year, and 5-year overall survival rates of ESCA patients. All findings are statistically significant (p value < 0.05).

Additionally, we utilized data from the TCGA database for Kaplan–Meier (KM) prognostic analysis in various subgroups. This analysis showed that increased GK expression significantly impacted overall survival (OS) and disease-specific survival (DSS) in ESCA patients, while its effect on progression-free interval (PFI) was not significant (Fig. 2A–C). Subgroup analysis revealed that high GK gene expression in patients with lymph node metastasis and those with a high BMI indicated a worse prognosis (Fig. 2D, E). Further subgroup details are available in Supplementary Fig. 1B.

### The potential diagnostic value of GK gene expression in patients with ESCA

In the TCGA database, we generated ROC (Receiver Operating Characteristic) curves and Nomogram plots for the GK gene to assess its diagnostic value. The analysis yielded an AUC (Area Under the Curve) of 0.897 (95% CI = 0.7831–0.962), indicating a high diagnostic accuracy (Fig. 2F). Additionally, Nomogram plots were created by integrating multiple clinical indicators (including T stage, N stage, M stage, gender, age, and GK expression) along with the results from the multifactorial regression analysis. These plots aimed to predict the survival rates of ESCA patients at 1, 3, and 5 years, with a higher point value representing a worse prognostic factor. Notably, the predictive score for prognosis based on GK gene expression level surpassed traditional indicators such as age, gender, and T-stage (Fig. 2G).

### Using clinical specimens to verify GK’s high expression in ESCA and assess its prognostic impacct

**Figure 3:**
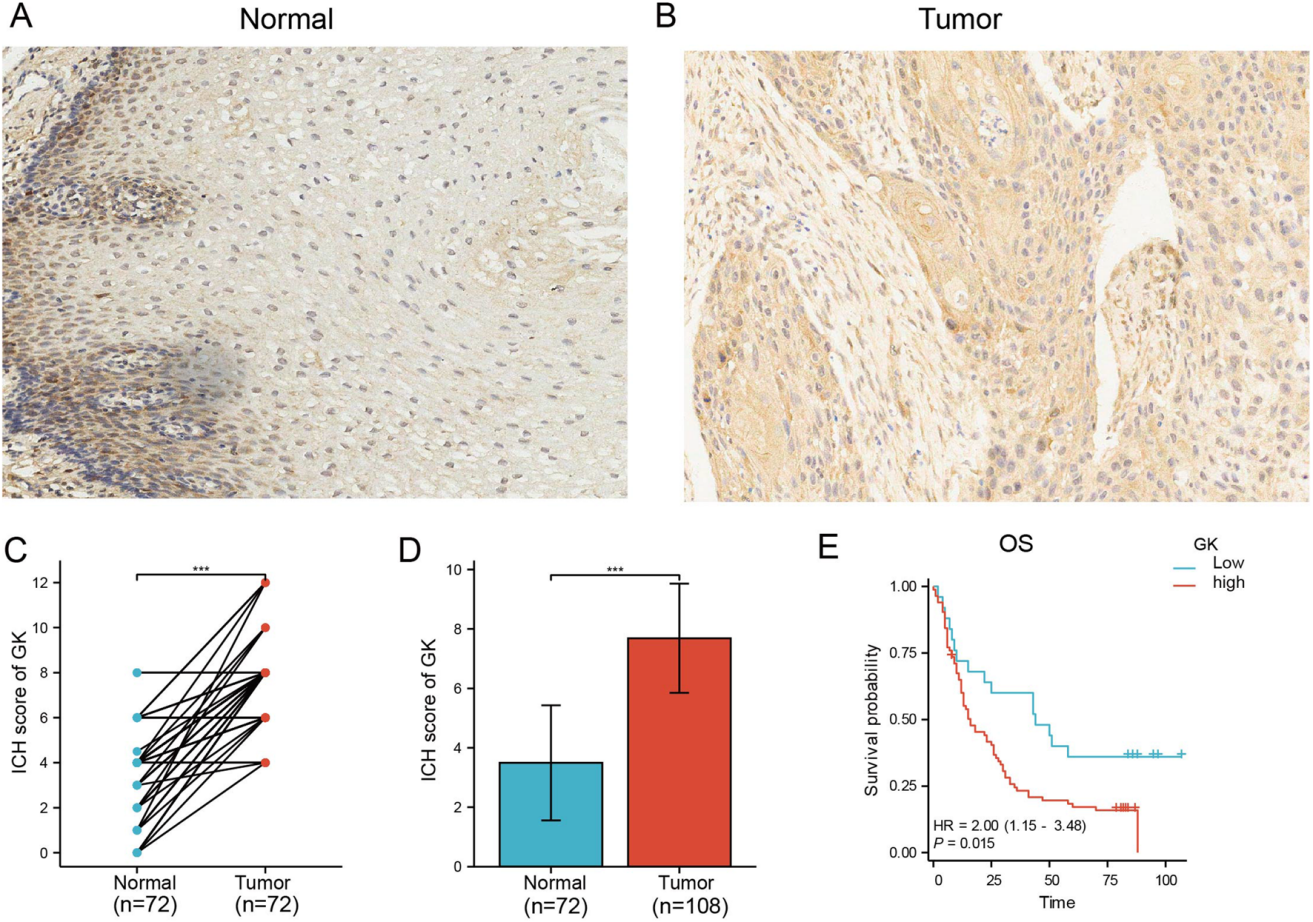
Protein expression of glycerol kinase (GK) in clinical ESCA tissues. (**A**, **B**) Immunohistochemical staining showcasing GK expression in normal tissues (**A**) and tumor tissues (**B**). (**C**) Paired analysis of GK expression in ESCA and normal tissues using clinical specimens. (**D**) Comparative analysis of GK expression in ESCA and normal tissues from clinical specimens. (**E**) Kaplan–Meier survival analysis depicting overall survival (OS) in clinical ESCA patients. Statistical significance is indicated as ***p < 0.001.

We used immunohistochemical staining to analyze 108 ESCA samples, including 10 at pathological stage I, 76 at stage II, and 22 at stage III. Additionally, 72 adjacent non-cancerous tissue samples served as controls (Fig. 3A, B). Both groups were subjected to immunohistochemical staining and evaluated using an integrative score. This analysis showed that 83 ESCA tissue samples had high GK expression, while 25 exhibited low expression. In the control group, 3 cases showed high expression, and 69 had low expression.

Further analysis included unpaired (p < 0.001, Fig. 3C) and paired sample assessments (p < 0.01, Fig. 3D), which consistently demonstrated significantly increased GK expression levels in ESCA patients. Additionally, KM survival analysis, integrating GK gene expression levels with patient survival time, indicated that high GK gene expression correlated with lower survival rates in ESCA patients (p = 0.015, Fig. 3E).

### Mutations of GK in ESCA

**Figure 4:**
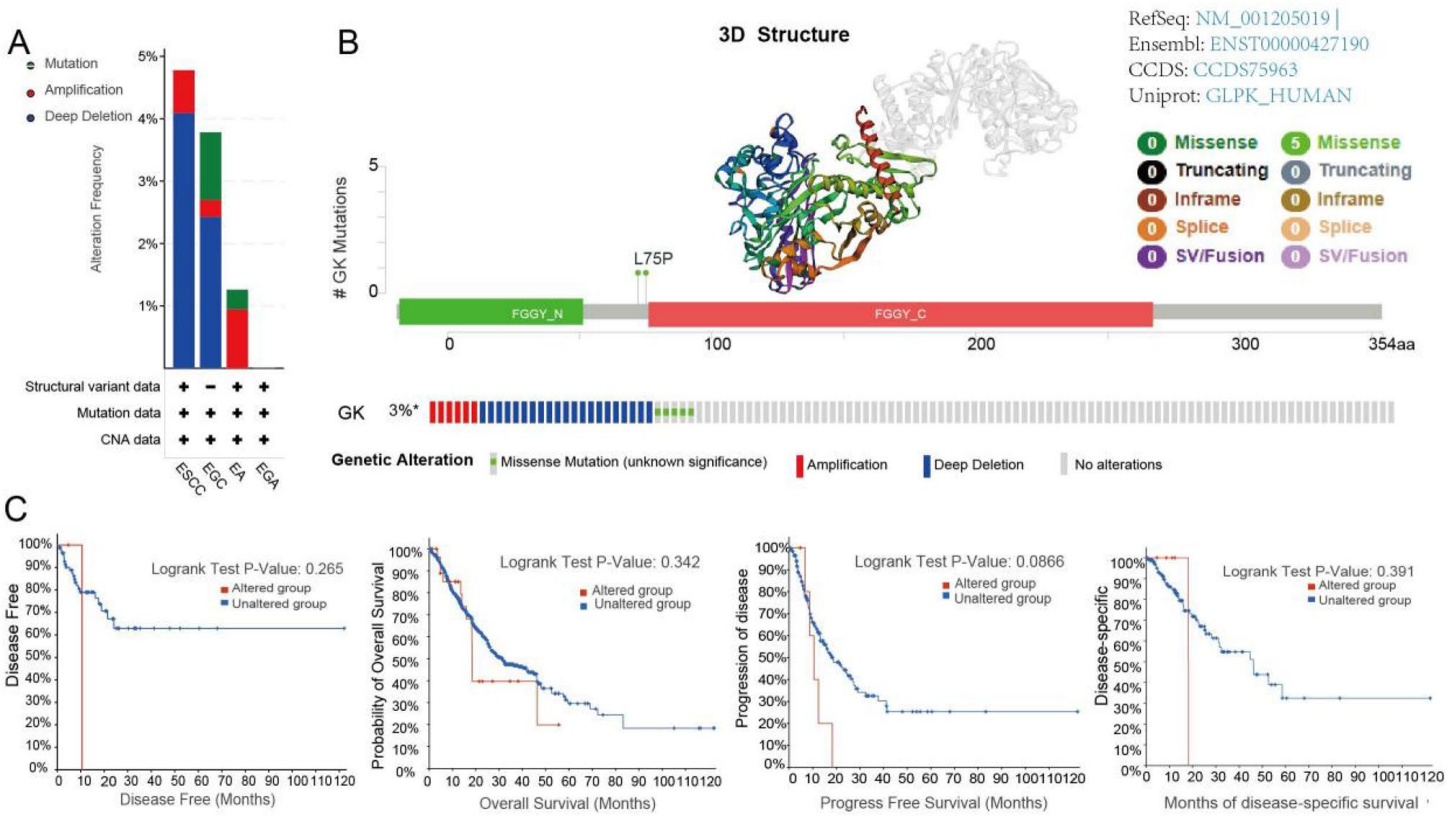
Mutation features of glycerol kinase (GK) in the TCGA-ESCA cohort. (**A**) The alteration frequency of GK across different pathological types of ESCA. (**B**) The alteration frequency of GK categorized by mutation type and site, accompanied by a 3D structure representation of the GK protein. (**C**) The association between GK mutation status and various clinical outcomes in ESCA patients, including disease-free probability, overall survival, disease progression, and disease-specific survival. Statistical significance is denoted as P value < 0.05.

In our study, we utilized the cBioPortal database to analyze the mutation of the GK gene in ESCA samples, incorporating various databases such as TCGA, MSK, ICGC, DGCI, UCLA, etc. Figure 4A illustrates the GK gene's mutation status across different pathological types of ESCA, with esophageal squamous cell carcinoma (ESCC) showing the highest mutation frequency at 4.78%. Additionally, we examined the mutation frequency, types of genetic alterations, mutation sites, and the number of cases (Fig. 4B). The overall mutation frequency of the GK gene was found to be 3%, and the identified genetic alterations included deep deletions, missense mutations, and amplifications.

Furthermore, we investigated the relationship between GK genetic alterations and patient prognosis. The analysis did not reveal a statistically significant association between GK genetic alterations and various prognostic measures, including Disease-Free Survival (p = 0.265), Overall Survival (p = 0.342), Progression-Free Survival (p = 0.0866), and Disease-Specific Survival months (p = 0.391). The results were similar between the groups with GK alterations and those without (Fig. 4C). These findings suggest that the mutation status of the GK gene may not have a significant impact on the prognosis of ESCA patients.

### Methylation of GK in ESCA

**Figure 5:**
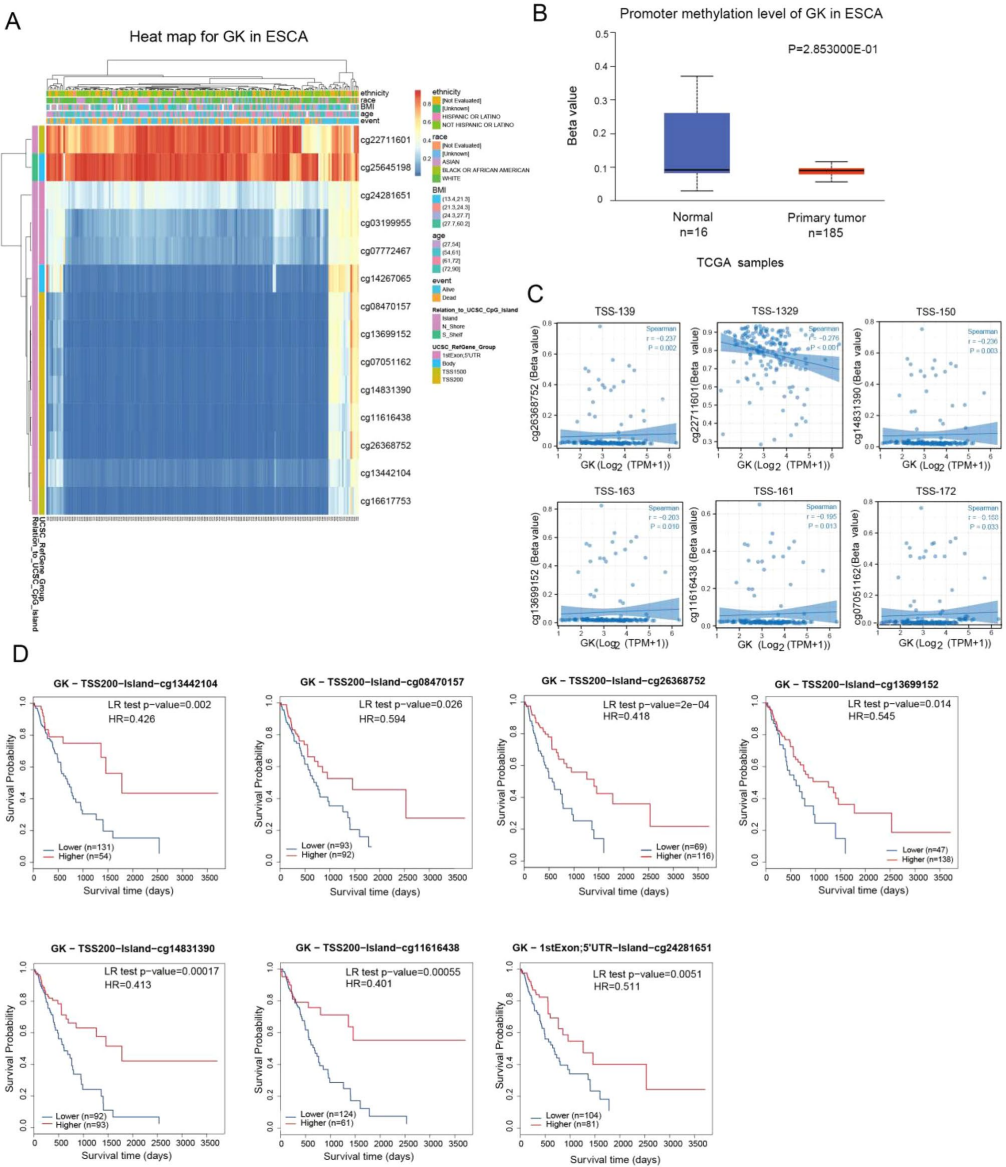
DNA methylation analysis of glycerol kinase (GK) using MethSurv. (**A**) Heatmap illustrating the methylation patterns of GK in ESCA patients. (**B**) Comparison of promoter methylation levels of GK between normal and tumor tissues in the TCGA-ESCA cohort. (**C**) Correlation between GK expression and methylation at specific CpG sites. (**D**) MethSurv analysis of the impact of varying methylation levels and sites on the survival time of ESCA patients.

Figure 5A depicts the methylation expression profile for each CpG site of the GK gene as derived from the TCGA database. In ESCA, the methylation level of GK, as analyzed by MethSurv, was found to be low (Fig. 5B). Additionally, there was a weak associated between GK gene expression and specific CpG sites, namely cg26368752, cg22711601, cg14831390, cg13699152, cg11616438, and cg07051162 (Fig. 5C).

MethSurv analysis further indicated that the overall survival rate of patients with hypomethylated GK was lower compared to those with hypermethylated GK, particularly at sites such as cg13442104, cg084770157, cg26368752, cg13699152, cg14831390, cg11616438, and cg24281651, all showing statistical significance (p < 0.05) (Fig. 5D). Additional CpG sites, which did not show statistically significant results, are detailed in Supplementary Fig. 1C. These results suggest that the methylation level of GK is a valid prognostic biomarker for ESCA.

### GK co-expression gene analysis: biological functions and pathway predictions

**Figure 6:**
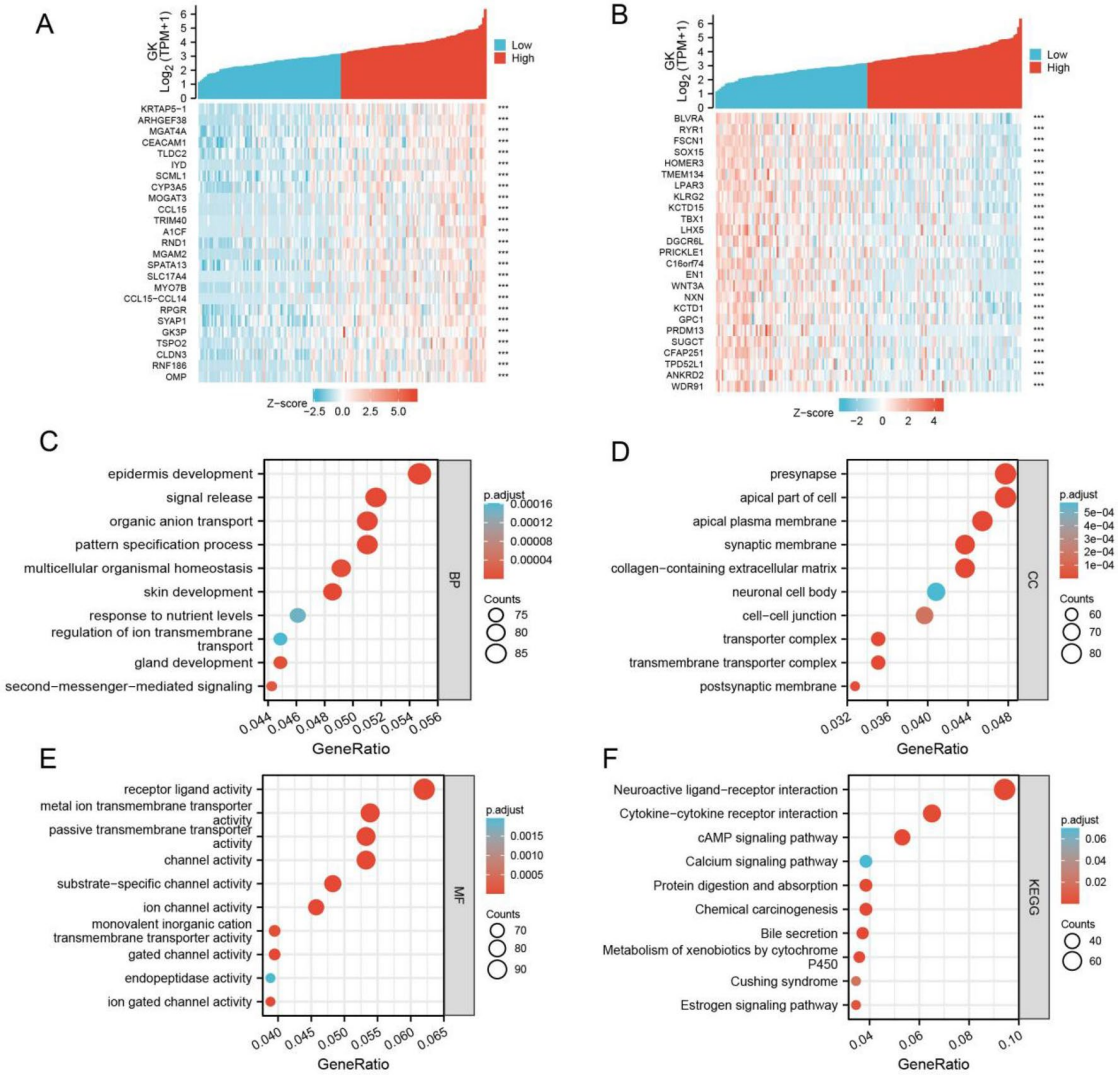
Glycerol kinase (GK) functional clustering and interaction network analysis of GK-related genes. (**A**) Heatmap displaying the top 25 genes in esophageal carcinoma (ESCA) positively correlated with GK. (**B**) Heatmap showing the top 25 genes in ESCA negatively correlated with GK. (**C-E**) Gene Ontology (GO) enrichment analysis illustrating the biological processes (BP) (**C**), cellular components (CC) (**D**), and molecular functions (MF) (**E**) associated with genes co-expressed with GK, based on the TCGA-ESCA cohort. (**F**) Kyoto Encyclopedia of Genes and Genomes (KEGG) enrichment analysis for genes co-expressed with GK.

In this study, data was extracted from the TCGA database to identify genes associated with GK expression. Utilizing the Spearman test, we identified the top 25 genes positively associated with GK expression and generated a heat map to visualize these associations (Fig. 6A). Similarly, another heat map was created for the top 25 genes negatively associated with GK expression (Fig. 6B).

To further explore the potential biological functions of these target genes, we conducted an enrichment analysis of genes co-expressed with GK (Log2FC absolute value > 1, P < 0.05). The analysis revealed significant enrichment in various biological processes (BP), cellular components (CC), molecular functions (MF), and KEGG pathways. Specifically, BP analysis showed significant enrichment in epidermis development, signal release, and organic anion transport (Fig. 6C). CC analysis indicated enrichment in presynapse, apical part of the cell, and apical plasma membrane (Fig. 6D). MF analysis highlighted enrichment in receptor ligand activity, metal ion transmembrane transporter activity, and passive transmembrane transporter activity (Fig. 6E). Finally, KEGG pathway analysis revealed significant enrichment in neuroactive ligand-receptor interaction, cytokine-cytokine receptor interaction, cAMP signaling pathway, and chemical carcinogenesis, among others (Fig. 6F).

**Figure 7:**
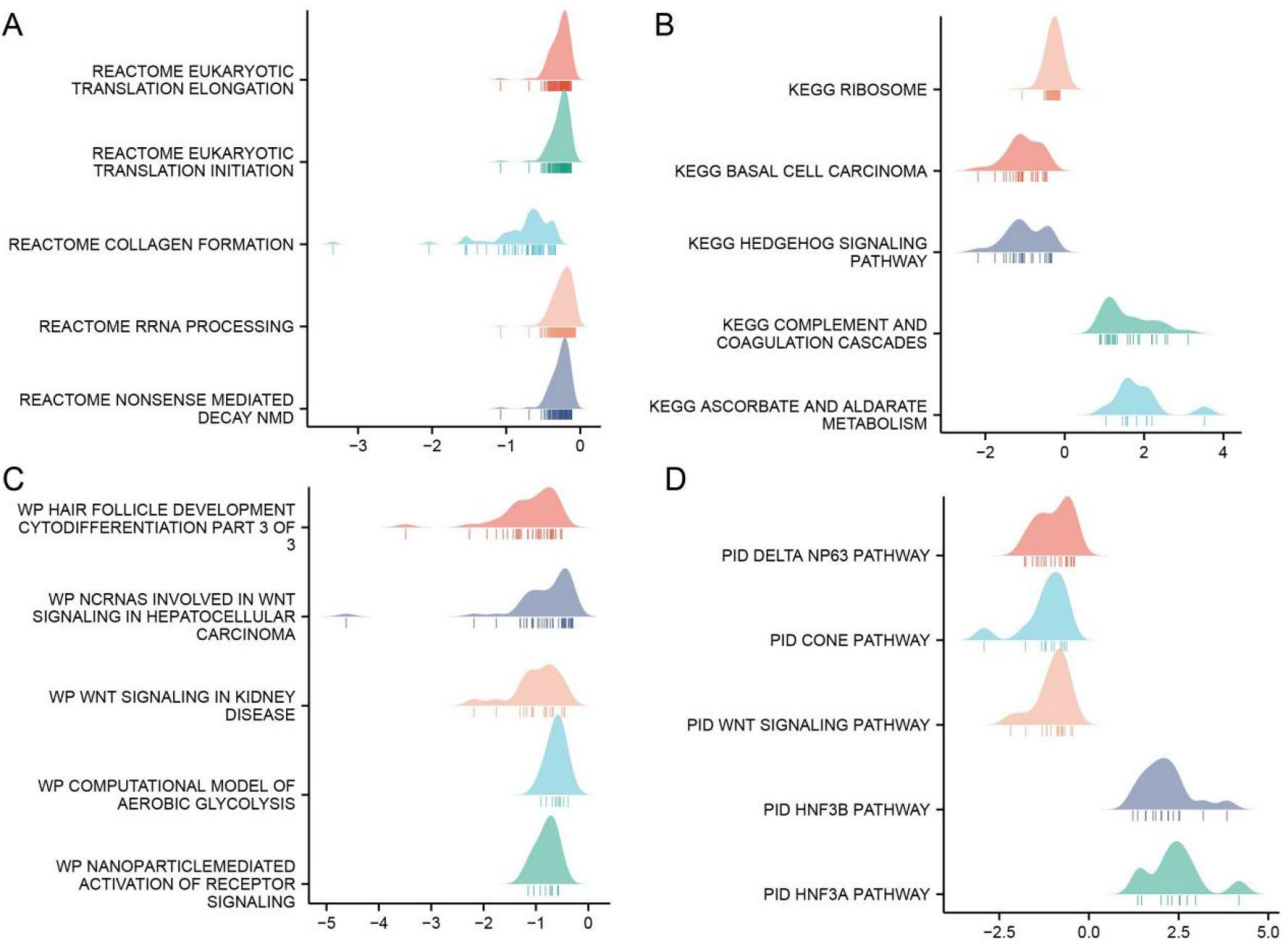
Significant gene set enrichment analysis results for glycerol kinase (GK), highlighting various pathway analyses. (**A**) Enrichment results in Reactome pathways. (**B**) Enrichment in Kyoto Encyclopedia of Genes and Genomes (KEGG) pathways. (**C**) Enrichment in WikiPathways (WP) pathways. (**D**) Enrichment in pathway interaction database (PID) pathways.

Additionally, we conducted Gene Set Enrichment Analysis (GSEA) and presented the findings in mountain plots (Fig. 7A–D). The GSEA results indicated enrichment in several key pathways, including REACTOME Eukaryotic Translation Elongation, KEGG Ribosome, KEGG Basal Cell Carcinoma, WikiPathways (WP) Ncrnas Involved in Wnt Signaling in Hepatocellular Carcinoma, PID Delta NP63 Pathway, and PID Wnt Signaling Pathway.

### Analysis of GK expression and immune infiltration

Immunotherapy has proven beneficial for many patients with ESCA, and our gene pathway analysis highlighted a significant enrichment in cytokine-cytokine receptor interactions, a pathway closely related to immunity. Consequently, we investigated the relationship between GK expression and immune infiltration in ESCA.

**Figure 8:**
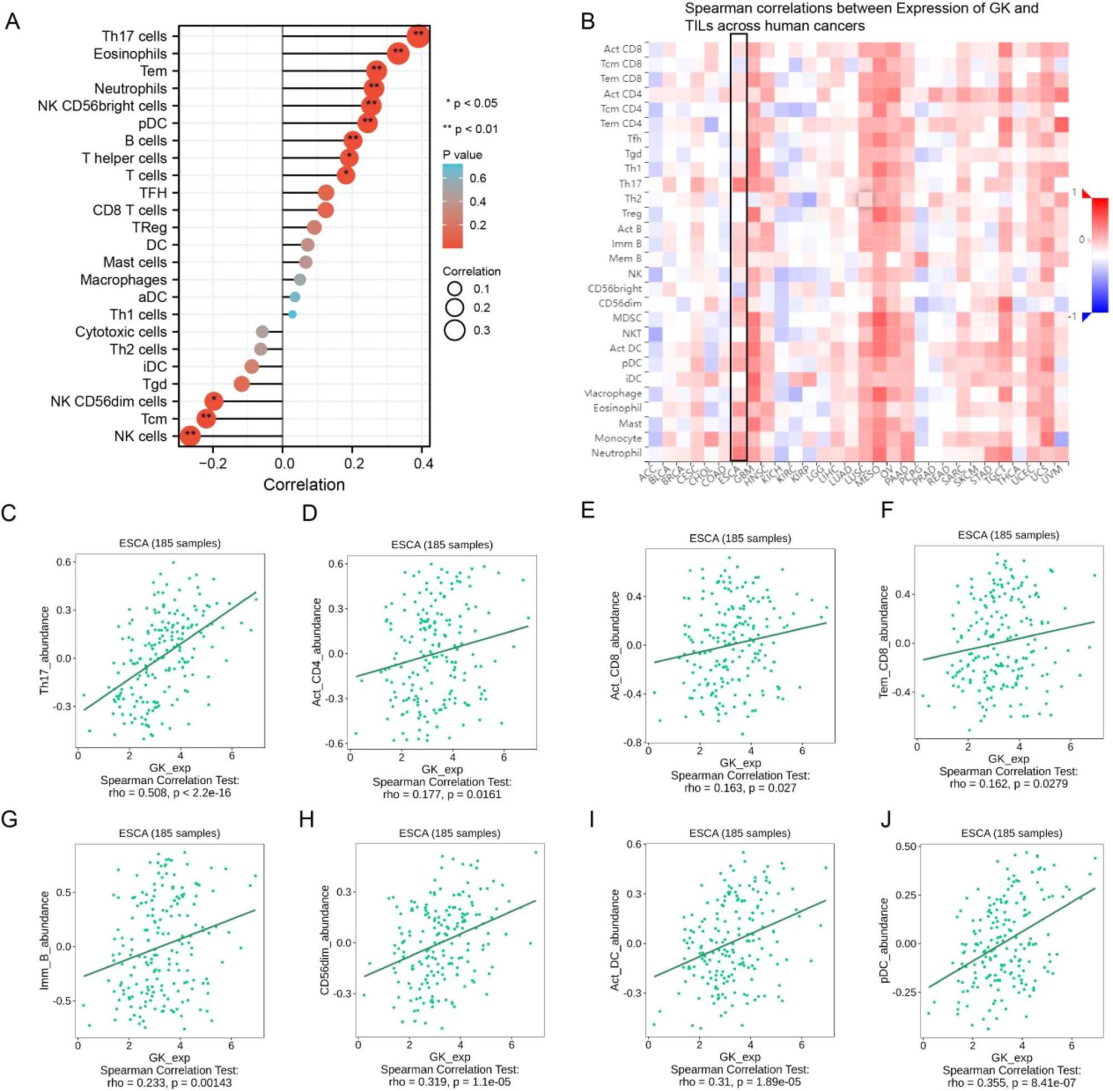
Correlation of tumor-infiltrating lymphocytes (TILs) with glycerol kinase (GK) gene expression in esophageal carcinoma (ESCA). (**A**) Lollipop plot depicting the differentiation in ratios of 24 immune cell types between ESCA tumor samples with high versus low GK expression. (**B**) Heatmap analysis illustrating the correlation between GK expression and TILs in tumor samples. (**C**–**J**) Scatter plots demonstrating the correlation of GK expression with eight types of TILs, specifically TH17 cells, Activated CD4 T cells (Act_CD4), Activated CD8 T cells (Act_CD8), T Effector Memory CD8 T cells (Tem_CD8), Immature B cells (Imm_B), CD56dim natural killer cells, Activated Dendritic Cells (ACT_DC), and Plasmacytoid Dendritic Cells (pDC).

Utilizing the TCGA dataset and the GSVA package (version 3.6.3), we assessed the level of immune cell infiltration in tumor tissues. Our analysis revealed a positive correlation between GK expression and various immune cells, including Th17 cells, eosinophils, effector memory T cells (Tem), neutrophils, NK CD56bright cells, plasmacytoid dendritic cells (pDC), T helper cells, and T cells. Conversely, a negative correlation was observed with natural killer (NK) cells and central memory T cells (Tcm) (Fig. 8A).

This finding was further corroborated using the TISIDB database (Fig. 8B), which confirmed significant correlations of GK expression with TH17 cells, activated CD4 and CD8 T cells, effector memory CD8 T cells, and other T lymphocytes, as well as with immune B cells, CD56dim cells, activated dendritic cells, and pDC (Figs. 8C–J). These results suggest a strong link between GK expression and tumor immune infiltration in ESCA.

**Figure 9:**
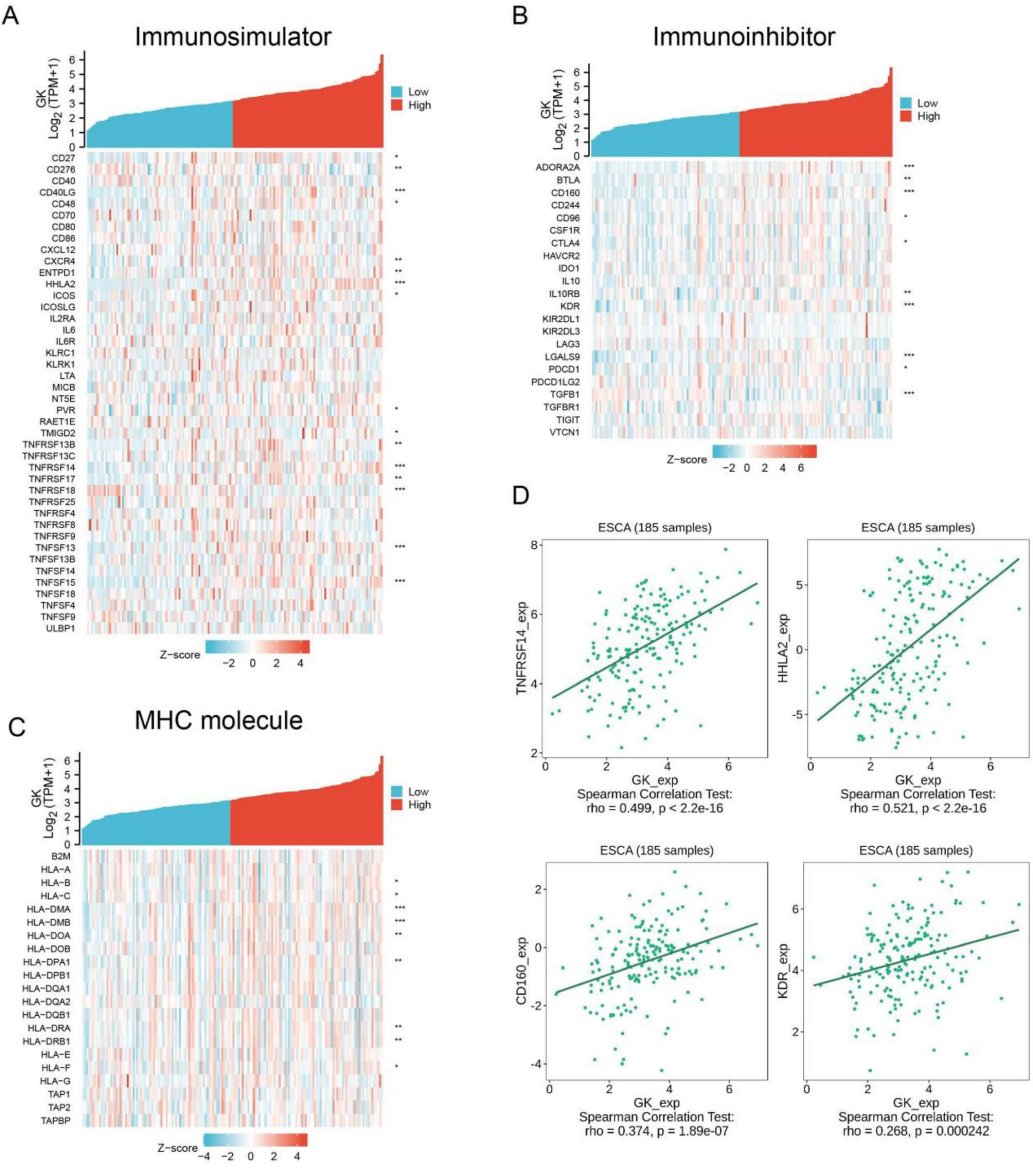
Correlation of immune modulators with glycerol kinase (GK) expression in esophageal carcinoma (ESCA). (**A**) Heatmap depicting the correlation between GK and immunostimulators. (**B**) Heatmap showing the correlation between GK and immunoinhibitors. (**C**) Heatmap illustrating the correlation between GK and Major Histocompatibility Complex (MHC) molecules. (**D**) Scatter plot demonstrating that GK expression is positively related to TNFRSF14, CD160, KDR, and HHLA2 in ESCA.

To delve deeper into the relationship between immunomodulatory sites and GK expression, we generated a heat map using data from the TCGA ESCA project. In the category of immunostimulators, we found significant associations between GK expression and various markers, including CD27, CD276, CD40LG, CD48, CXCR4, ENTPD1, HHLA2, TNFRSF13B, TNFRSF14 (Fig. 9A). For immunoinhibitors, significant associations were observed with GK expression and ADORA2A, BTLA, CD160, CD96, CTLA4, IL10RB, KDR, among others **(**Fig. 9B). In the MHC molecule category, GK expression showed close associations with HLA-A, HLA-B, HLA-DMA (Fig. 9C). Finally, in the analysis of various immunomodulatory points, including TNFRSF14, HHLA2, CD160, KDR, GK demonstrated a significant positive correlation (Fig. 9D).

## Discussion

In this study, we present pioneering evidence linking high Glycerol Kinase (GK) expression to the incidence of Esophageal Squamous Cell Carcinoma (ESCA), encompassing both Esophageal Adenocarcinoma (EA) and ESCC. Our comprehensive analysis, leveraging data from TIMER, TCGA, GEO databases, and clinical specimens, reveals a consistent pattern: GK expression is significantly higher in ESCA than in adjacent tissues. This finding suggests GK’s potential as a novel biomarker for ESCA diagnosis and prognosis.

A crucial aspect of our research involved examining the relationship between GK expression and various ESCA facets, such as progression, mutation, methylation, and immune cell infiltration. Notably, we observed a significant association between GK expression and the activity of T and B lymphocytes, indicating GK’s possible role in tumor immune infiltration. Furthermore, our findings indicate that elevated GK expression correlates with poorer patient prognosis, emphasizing the need for further exploration into GK's impact on lymph node metastasis and ESCA progression. These insights position GK as a potential novel biomarker for the diagnosis and prognosis of ESCA, applicable as an early biomarker or a target for new preventative strategies.

Research on GK in cancer has been limited. A study on preneoplastic lesions in a rat model of liver cancer reported a significant reduction in GK expression compared to adjacent non-preneoplastic tissue^19^. This decrease in GK protein within preneoplastic lesions suggests that glycerol uptake and metabolism in the early stages of liver cancer development are predominantly functions of the surrounding tissue, not the lesion itself. Reduced GK protein expression diminishes the capability to import glycerol and convert it into G3P, potentially protecting precancerous hepatocytes from the harmful effects of G3P within the lesion. This may influence the growth of precancerous hepatocytes, leading to cell cycle arrest and apoptosis^19,20^. Sriram’s investigation into glycerol kinase overexpression in rat hepatoma cells sheds light on how GK upregulation alters cellular metabolism, with a particular emphasis on glycerol and lipid metabolic pathways^21^. Conversely, database data indicate increased GK expression in breast and gastric cancers, but a decrease in hepatocellular carcinoma and renal papillary cell carcinoma, implying a complex, dual role of GK in cancer development^15^.

Our analysis of the TCGA database reveals a disparity in GK expression: it is higher in patients with lymph node metastasis but does not significantly vary across different T stages. This inconsistency highlights the need for further experimental validation of GK’s role. Future research should include comprehensive experiments to validate GK’s function in ESCA, involving both cellular and animal models. Additionally, KM analysis^12^, database studies, and clinical data show that higher GK expression in ESCA patients correlates with poorer prognosis and increased risk of lymph node metastasis. An analysis of human liver cancer samples from the TCGA, using KM survival plots, suggests that low GK and GPDH expressions are associated with reduced overall survival^22^. Given the importance of lymph node metastasis in esophageal cancer progression^10^, further experiments are essential to determine whether GK influences the prognosis of esophageal cancer through lymph node metastasis.

There are three GK variants: GK, GK2, and GK5^23^. Glycerol Kinase 5 (GK5) is implicated in several processes, including the glycerol metabolic process. Notably, exosomal mRNA of GK5 in the plasma of patients with gefitinib-resistant adenocarcinoma is significantly higher than in gefitinib-sensitive patients. GK5 mRNA and protein levels in gefitinib-resistant human lung adenocarcinoma cells (PC9R and H1975) are also significantly upregulated.8 Silencing GK5 in PC9R cells induces mitochondrial damage, caspase activation, cell cycle arrest, and apoptosis through the Sterol regulatory element-binding protein 1/Stearoyl-CoA-desaturase 1(SREBP1/SCD1) signaling pathway. This suggests that GK5 plays a role in the development of gefitinib resistance mechanisms^24,25^. The potential for GK to induce resistance to anti-tumor drugs in ESCA necessitates further study to understand its mechanism of action.

As a key enzyme in glycerol metabolism and an intermediary between carbohydrate and fat metabolism, GK’s significance extends beyond its primary metabolic role^4,5^. Recent advancements in cancer genome sequencing have shed more light on the relationship between lipid metabolism genes and tumor pathogenesis^9,26,27^. Research by Lilly S. Parr indicates that GK functions multifariously at the interface of carbohydrate and fat metabolism^4^, with the ATP-stimulated translocation promoter (ASTP) function of GK playing a more substantial role than its enzymatic activity in adipogenesis. Previous studies have highlighted GK’s multiple functions, providing insights into glycerol kinase's diverse protein functions^28,29^, but have not explored its direct correlation with tumor pathogenesis or development.

Given these findings, we hypothesize that GK may be involved in the regulation of ESCA onset, invasion, and metastasis, serving as a potential diagnostic marker and therapeutic target. In our study, all pathological types were esophageal squamous cell carcinoma; however, TCGA data included both EA and ESCC, suggesting potential variability in results. With EA's increasing incidence in China^30^, larger sample sizes, encompassing both EA and ESCC, are crucial to further substantiate GK's role in esophageal cancer. Moreover, the specific pathways and functions of GK expression in ESCA require further elucidation through future cell and animal experiments. We recognize the inherent limitations of solely relying on online tools and databases such as TCGA, which are subject to continuous updates. Future studies will focus on incorporating more dynamic and comprehensive data sources to validate our findings.

## Material and methods

### GK gene expression analysis

We obtained mRNA expression data from the TCGA database (https://cancergenome.nih.gov), including 162 ESCA and 11 adjacent non-tumor samples, with corresponding clinicopathological information. Additionally, TCGA_GTEx database data with 182 ESCA and 666 non-tumor esophageal samples were selected. We analyzed the differences in GK mRNA expression between tumor and normal tissues. Furthermore, the GSE161533 gene expression profile, comprising 28 ESCA samples and 28 adjacent non-tumor samples, was downloaded from GEO (https://www.ncbi.nlm.nih.gov/geo/) for result validation. Clinical ESCA specimens were used for experimental confirmation.

### Survival analysis

To assess the impact of GK gene expression on the survival rates of ESCA patients with varying clinical features, hazard ratios (HR) and log-rank p-values within a 95% confidence interval were computed using KM plotter (http://kmplot.com/analysis/). For receiver operating characteristic (ROC) analysis, we employed the pROC package (version 3.6.3) to determine the area under the curve (AUC) values. Cox regression analysis was conducted to identify independent prognostic factors. Additionally, we developed nomogram plots to estimate the 1, 3, and 5-year overall survival probabilities for ESCA patients.

### Tissue microarray

Human ESCA tissue microarrays, comprising 108 ESCA and 72 adjacent non-tumor samples, were sourced from Shanghai Outdo Biotech Company. The clinical cohort included 82 males and 26 females, aged between 29 and 84 years, with an average age of 65.5. These patients underwent surgery between July 2006 and December 2008, and were followed up until July 2015, with a follow-up duration ranging from 6.6 to 9 years.

### Hub gene enrichment analysis

To elucidate GK’s biological role in ESCA, we explored the GEPIA database (http://gepia.cancer-pku.cn/) to identify genes associated with GK^13^. Gene ontology (GO) analysis was employed to classify the biological processes (BPs), cellular components (CCs), and molecular functions (MFs) related to these genes. Furthermore, the Kyoto Encyclopedia of Genes and Genomes (KEGG) pathway analysis was utilized to examine their functional pathways. Using Gene Set Enrichment Analysis (GSEA), we assessed whether there was a significant variation in the functionally annotated genes across ESCA samples with differing levels of GK expression, aiming to uncover potential molecular mechanisms of these target genes^14^.

### Timer database analysis

In our research, we utilized the Tumor Immune Estimate Resource (TIMER, http://timer.cistrome.org), an online database offering extensive analysis and visualization tools for tumor-infiltrating immune cells^15^. The TIMER database was employed to analyze the variations in the abundance of GK genes between tumor and normal tissues, facilitating the exploration of immunological, clinical, and genomic characteristics of ESCA.

### Immune cell infiltration analysis

The interplay between tumorigenesis, progression, treatment, and the immune system is vital. Utilizing data from the TCGA dataset, we employed the GSVA package (version 3.6.3) to assess immune cell infiltration levels in tumor tissues. Additionally, the TISIDB database (http://cis.hku.hk/TISIDB/) was used to further validate and analyze interactions between tumors and immune cells^16^. Our study focused on exploring the relationship between the GK gene and various immune factors, including immunostimulators, immunoinhibitors, and MHC molecules. All statistical analyses were conducted using Spearman's test.

### cBioPortal database analysis

The cBioPortal database (https://www.cbioportal.org/) serves as an open platform for examining multidimensional cancer genomic data^17^. In our study, it was utilized to investigate the types and frequencies of mutations in GK genes specific to ESCA. Additionally, the database’s survival module analysis enabled us to perform Overall Survival (OS), Progression-Free Survival (PFS), and Disease-Specific Survival (DSS) analyses for GK genes.

### Methylation analysis

Abnormal DNA methylation plays a significant role in tumor development. The Methsurv website (https://biit.cs.ut.ee/methsurv/) is a useful tool for analyzing DNA methylation of the GK gene at CpG sites and its prognostic implications^18^.

### Immunohistochemistry staining

The ESCA tissue microarrays were heated at 63 °C for 1 h for wax melting. Post-baking, they underwent dewaxing and antigen retrieval. The microarrays were then washed in PBS buffer, followed by the addition of GK antibody (supplied by Hua’an Biological Co., Ltd.) at a dilution of 1:300, and incubated in a low-temperature refrigerator. Subsequently, diaminobenzidine (DAB) solution was used for color development, and Harris hematoxylin served as the counterstain. After mounting, pathologists assessed the slides, rating cell staining on a scale: 0 for no staining, 1 for weak positivity, 2 for moderate positivity, and 3 for strong positivity. This was combined with a score for positive cell density for GK staining, where 1 represented ≤ 25% positive cells, 2 for 26–50%, 3 for 51–75%, and 4 for 76–100%. The product of these two scores determined the final result, categorizing 0–6 as low expression and 7–12 as high expression.

### Statistically analysis

Bioinformatics analysis was conducted using R (Version 3.6.3). We employed various statistical methods, including t-tests, chi-square tests, logistic regression analysis, and log-rank tests for p-values. The prognostic significance of GK was evaluated using the Kaplan–Meier method, along with univariate and multifactor COX regression. In our analyses, the significance levels were denoted as follows: “for p < 0.05,” for p < 0.01, and “for p < 0.001”.

### Ethics declarations

This retrospective study was performed in accordance with the Declaration of Helsinki. Approval was obtained from the Institutional Research Ethics Committe of Xianju county People’s Hospital(protocol 2022-065). The Institutional Research Ethics Committe of Xianju county People’s Hospital waived the need of informed consent due to this study’s retrospective nature and the anonymized processing of patient data.

## Conclusion

In summary, our findings indicate a significant association between elevated GK expression and the incidence of ESCA, potentially correlating with poor prognosis, notably in terms of reduced Overall Survival (OS) and Disease-Specific Survival (DSS), as well as increased immune cell infiltration. Multivariate regression analysis identified GK expression as an independent risk factor influencing ESCA prognosis. ROC analyses and Nomogram plots further demonstrated GK's efficacy as a predictive biomarker for distinguishing between ESCA and normal tissue. These results suggest that GK could serve as a valuable independent prognostic biomarker and a potential target for therapeutic intervention.

### Supplementary Information

Supplementary Figure 1.

## Data Availability

The study's original data is included in the article and its supplementary files. For further inquiries, please contact the corresponding author.
