# Supplementary material for: Glycerol kinase enzyme is a prognostic predictor in esophageal carcinoma and is associated with immune cell infiltration
Source: Sci Rep. 2024 Feb 16;14:3922. doi: 10.1038/s41598-024-54425-x (PMC10873286; doi:10.1038/s41598-024-54425-x)
Supplement: Supplementary file 1 — Supplementary Figure 1. [file 41598_2024_54425_MOESM1_ESM.docx]

**Supplementary figure**


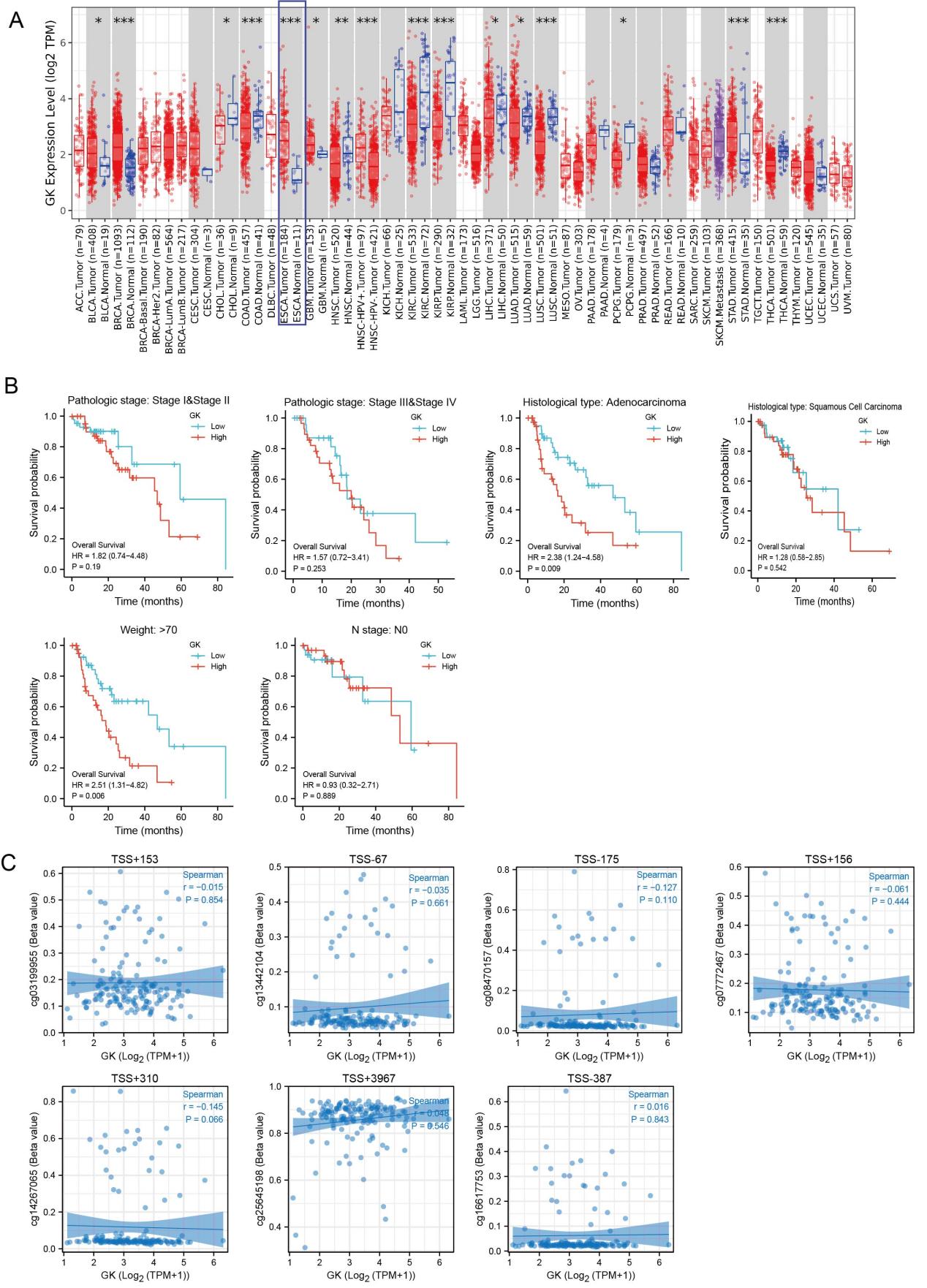


**Supplementary figure 1** (**A**) Glycerol Kinase (GK) expression levels across various cancer types, sourced from the TIMER database. (**B**) Subgroup Kaplan-Meier survival curve analysis based on the TCGA-ESCA cohort. (**C**) Correlation between GK expression and specific CpG sites.
